# Leaf litter and fine roots have distinct effects on particulate and mineral‐associated soil organic matter in a tree common garden

**DOI:** 10.1111/nph.70854

**Published:** 2026-01-05

**Authors:** Ashley Lang, Rachel A. King, Jamie Pullen, Catherine Fahey, John D. Parker, Richard P. Phillips

**Affiliations:** ^1^ Department of Biology Indiana University Bloomington IN 47405 USA; ^2^ Smithsonian Environmental Research Center Edgewater MD 21037 USA

**Keywords:** common garden, fine roots, isotopes, leaf litter, mineral‐associated organic matter, mycorrhizal fungi, particulate organic matter, soil carbon

## Abstract

- Soil organic matter (SOM) is primarily derived from leaf and root inputs, but the relative contributions of each are difficult to study without the use of isotopic tracers. Furthermore, associations between trees and mycorrhizal fungi can influence the production and persistence of SOM.
- We quantified tree inputs and carbon and nitrogen content of three SOM fractions – free and occluded particulate organic matter (fPOM and oPOM, respectively), and mineral‐associated organic matter (MAOM) – in a tree common garden in Maryland, USA, where the trees and soil have distinct isotopic signatures due to prior use of the land as a cornfield.
- We found that stem basal area was not associated with concentrations of carbon (C) in any SOM pool but that fine root biomass was positively related to the proportion of tree‐derived MAOM‐C. POM was positively associated with leaf litter carbon : nitrogen ratio (C : N), but tree‐derived MAOM was not associated with fine root C : N. Tree mycorrhizal type did not influence the relative importance of leaf and root‐derived SOM.
- Our results indicate that leaf litter and fine roots have distinct roles on POM and MAOM formation, respectively, and that these effects are consistent across tree mycorrhizal associations in early stand development.

## Introduction

The dynamics of particulate organic matter (POM) and mineral‐associated organic matter (MAOM) have the potential to influence long‐term patterns in soil carbon (C) sequestration. The structural nature of the organomineral associations in MAOM can inhibit access to microbial enzymes, making it less susceptible to decomposition than POM (Baldock & Skjemstad, 2000). Therefore, MAOM represents a relatively persistent pool of soil C that may be insensitive to changes in climate or land use (Torn *et al*., 1997; Heckman *et al*., 2022). POM, however, may be more tractable to manage for soil C sequestration, and, as it is not limited by the availability of reactive mineral surfaces, can potentially accumulate indefinitely (Angst *et al*., 2023). Thus, understanding the conditions impacting both POM and MAOM formation is an important step in predicting future soil C sequestration (Lavallee *et al*., 2019).

The formation and persistence of POM and MAOM are controlled by a variety of interrelated ecosystem properties. These properties may include the direct components of POM and MAOM – the characteristics of the organic matter and available soil minerals – or conditions that influence the environment for POM and MAOM formation and decomposition, including climate (Córdova *et al*., 2018; Haddix *et al*., 2020; Heckman *et al*., 2023; Lang *et al*., 2023). Organic matter characteristics, soil mineralogy, and climate are difficult to study in isolation because they often covary, but each may have specific impacts on the formation of POM and MAOM.

Chemical and physical characteristics of organic inputs influence the distribution of soil organic matter (SOM) into particulate and mineral‐associated forms (Cotrufo *et al*., 2013; Robertson *et al*., 2018; Lavallee *et al*., 2019). Generally, past work has shown that leaf and root litter containing more complex molecules, including lignin and structural carbohydrates, has a longer residence time in the soil as POM, whereas labile leaf and root litter stimulates microbial activity, producing microbial exudates and necromass that become stabilized on minerals and form MAOM (Cotrufo *et al*., 2013; Córdova *et al*., 2018; but see Elias *et al*., 2024). Furthermore, the physical location of the organic matter inputs to soil can affect the likelihood it will form mineral associations (Sokol *et al*., 2018). Generally, belowground inputs such as decaying roots or mycorrhizal fungi and rhizodeposits are expected to promote more efficient formation of MAOM than leaf litter due to their proximity to soil minerals and their preferred use as microbial substrates (Sokol & Bradford, 2019).

Furthermore, mycorrhizal associations may be a useful predictor of a tree species' impact on SOM pools (Phillips *et al*., 2013; Frey, 2019). Associations with arbuscular mycorrhizal (AM) fungi, or ectomycorrhizal (ECM) fungi, are often correlated with tree traits that influence organic matter inputs to soil, including the production, chemistry, and fate of both above‐ and belowground tissues. For example, temperate trees that associate with AM fungi typically have higher quality leaf litter than those associated with ECM fungi (Cornelissen *et al*., 2001; Keller & Phillips, 2019). Furthermore, AM and ECM associations can influence root dynamics (Valverde‐Barrantes *et al*., 2017; See *et al*., 2019), the quality of SOM inputs through mycelial turnover and rhizodeposition (Craig *et al*., 2018; Keller *et al*., 2021; Huang *et al*., 2022), and the within‐tree allocation of biomass to leaf and root tissue (Jevon & Lang, 2022). Given the direct links between these processes and the formation of POM and MAOM, tree mycorrhizal associations may be a useful predictor of a tree species' impact on SOM pools.

The uncertainty surrounding the effects of organic matter properties on the formation of MAOM vs POM limits our understanding of individual tree species impacts on soil C stocks. Furthermore, impending shifts in forest species composition across the temperate zone are likely to alter the quality and quantity of organic matter inputs to soil (Craig *et al*., 2018; Jo *et al*., 2019); therefore, a complete understanding of the impacts of organic matter characteristics on POM and MAOM formation is critical for predicting future soil C stocks in temperate forests. Common gardens enable us to test the importance of plant traits on SOM dynamics *in situ*, in which the soil properties and climate are consistent across experimental units and species effects on SOM can be assessed independently. Here, we aim to improve our understanding of how the quality and quantity of above‐ and belowground plant inputs influence the formation of MAOM vs POM, and examine whether these effects are consistent across tree species with different mycorrhizal associations. We conducted this work at the Smithsonian Environmental Research Center's BiodiversiTREE research site, a common garden of plots planted with either 1, 4, or 12 temperate tree species. The site is uniquely suited for studies of MAOM formation from recent tree inputs, as it was formerly planted with corn, a C_4_ crop, for at least three decades (Devaney *et al*., 2020), giving the soil a higher baseline C isotope ratio (i.e. δ^13^C) than soils used to grow C_3_ plants. This distinction allowed us to estimate the relative quantity of MAOM‐C derived from the (C_3_) planted trees and the pre‐existing MAOM‐C at the time of planting.

We assessed the relationships between aboveground factors (leaf litter carbon : nitrogen ratio (C : N) and tree basal area) and belowground factors (fine root C : N and biomass) on SOM fractions in plots containing single tree species and mixtures of species. First, we expected the total concentration of soil C in each SOM fraction and the fraction of tree‐derived MAOM‐C to be higher in plots with greater productivity because fast‐growing trees should have greater litter inputs to soil. We also expected that litter with higher C : N would promote the formation of POM‐C, rather than MAOM‐C, due to slower decomposition. Furthermore, because belowground inputs are more closely associated with MAOM formation in experimental conditions, we expected that root litter chemistry would exert a stronger influence on MAOM‐C concentrations, whereas leaf litter chemistry would more directly influence POM‐C concentrations. Lastly, we expected tree mycorrhizal association would be associated with metrics of leaf and root litter chemistry, thus acting as a secondary predictor of POM and MAOM‐C concentrations in soil.

## Materials and Methods

### BiodiversiTREE site description

**Fig. 1:**
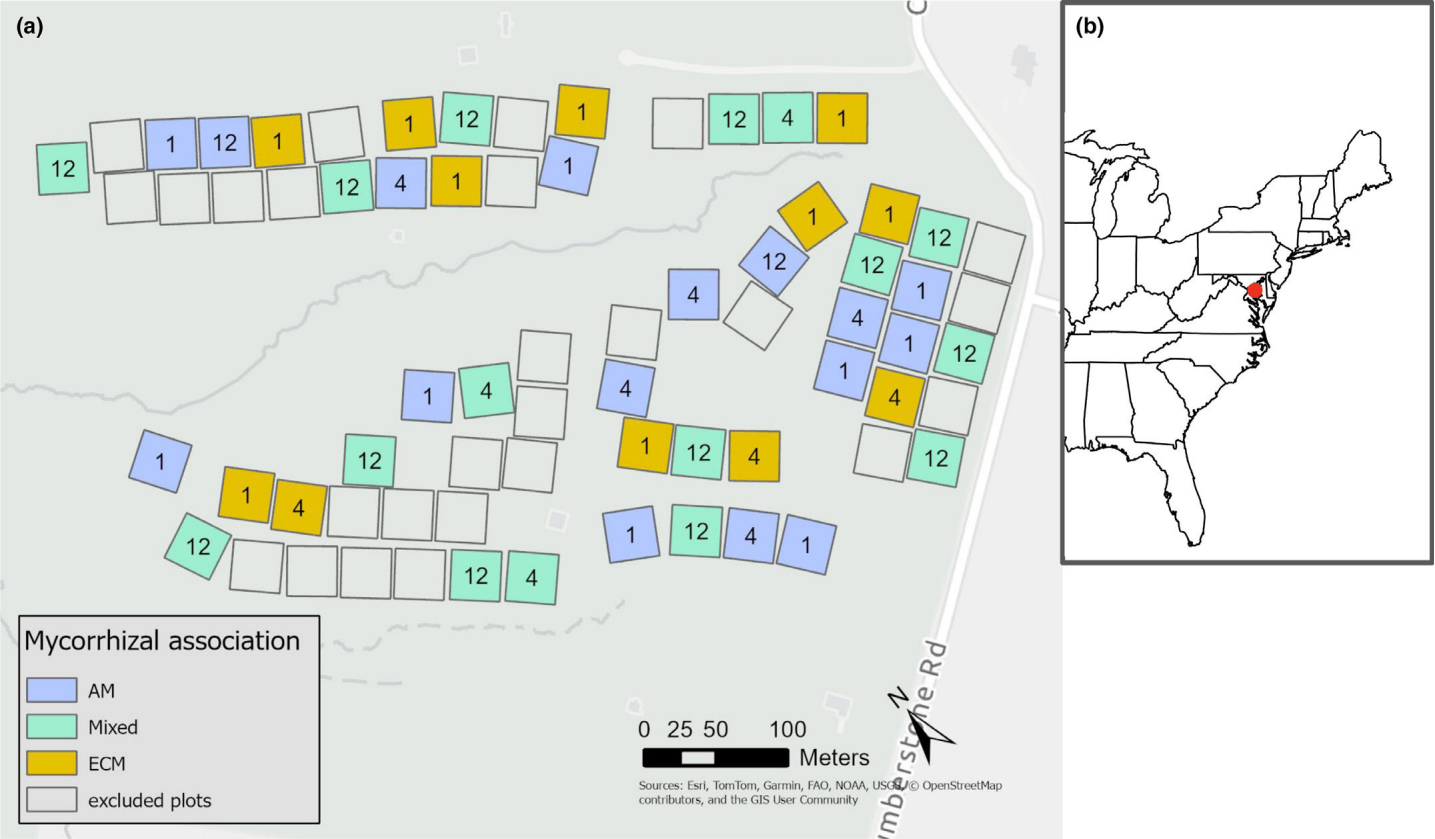
Site design and geographic context. (a) BiodiversiTREE plots used in this study. Plots dominated by arbuscular mycorrhizal (AM) tree species highlighted in blue, mixed plots in green, and plots dominated by ectomycorrhizal (ECM) trees in yellow. Numbers indicate the number of tree species planted in each plot. Plot mycorrhizal associations were determined by basal area percentage, with AM and ECM plots having > 90% basal area from AM and ECM‐associated trees, respectively; plots containing < 90% basal area of trees from each mycorrhizal type were considered mixed. (b) Location of study site within the mid‐Atlantic region of the eastern United States.

Our study was conducted at the BiodiversiTREE experimental site at the Smithsonian Environmental Research Center in Edgewater Maryland (38°52′7″N, 76°33′6″W). BiodiversiTREE consists of 70 planted plots and is part of TreeDivNet, a global network of tree diversity experiments (Paquette *et al*., 2018). The plots, established in 2013, were planted on fields formerly used to grow corn and contain a mix of locally common tree species planted in combinations of either 1, 4, or 12 species (Fig. 1). Soils in the study area are mesic Typic Hapludults derived from loamy fluviomarine deposits (Nachtergaele, 2001). We selected a subset of 43 plots to sample; selections were based on minimizing plots with high sapling mortality (King *et al*., 2023) and representing a range of species both in monocultures and in mixed‐species plots. Nine species, in particular, constituted the majority of the biomass in the plots we studied: *Carpinus caroliniana* Walt., *Cornus florida* L., *Fagus grandifolia* Ehrh. Little, *Liquidambar stryaciflua* L., *Liriodendron tulipifera* L., *Platanus occidentalis* L., *Quercus alba* L., *Quercus pagoda* Raf., and *Quercus rubra* L. The mycorrhizal associations of each tree species (Table 1) were determined by using the FungalRoot database (Soudzilovskaia *et al*., 2020).

**Table 1:** Mycorrhizal association, mean leaf litter carbon : nitrogen ratio (C : N), and fine root C : N of samples collected from the nine most abundant tree species in the study plots.

Mycorrhizal association	Species	Leaf litter C : N	Fine root C : N
		Mean (SD)	Mean (SD)
AM	*Cornus florida* L.	71.8 (na)	35.0 (7.9)
	*Liquidambar styraciflua* L.	94.9 (22.1)	38.8 (3.8)
	*Liriodendron tulipifera* L.	51.9 (2.9)	33.3 (5.8)
	*Platanus occidentalis* L.	61.1 (7.5)	42.7 (2.6)
ECM	*Carpinus caroliniana* Walt.	46.9 (7.8)	41.2 (8.2)
	*Fagus grandifolia* L.	65.0 (10.6)	33.1 (4.6)
	*Quercus alba* L.	61.1 (4.4)	43.3 (9.3)
	*Quercus pagoda* Raf.	56.9 (10.2)	56.3 (14.4)
	*Quercus rubra* L.	53.0 (5.1)	33.4 (2.5)

Freshly fallen leaf litter samples were collected from four trees of each species (*n* = 4 samples per species). Root samples were isolated from soil (0–5 cm depth) collected in the center of each single‐species plot (*n* = 2 plots per species). Mycorrhizal designations determined using genus classifications from the FungalRoot database (Soudzilovskaia *et al*., 2020). AM, arbuscular mycorrhizal; ECM, ectomycorrhizal; na, not applicable.

### Sample collection and analysis

Freshly fallen leaves were collected from the base of a tree at the center of a subset of monoculture plots in fall 2020. A leaf sample was collected from four plots per species, with the exception of *C. florida*, which was collected from one plot due to sampling constraints (*n* = 33 leaf litter samples). Leaves were dried at 60°C and the entire sample, including petioles, was ground and homogenized for elemental analysis.

Soil samples were collected in fall 2020 near the center of each plot with a 2.54‐cm‐diameter soil corer. Samples were collected from 0 to 5 cm and subdivided into two depths: 0–2 and 2–5 cm. Samples were passed through a 2‐mm mesh sieve to remove coarse materials; fine roots (< 2 mm diameter) were carefully removed with tweezers. Fine roots were then rinsed, oven‐dried, weighed, and set aside for chemical analysis. Each soil sample was oven‐dried, and a 2 g subsample was set aside for density fractionation.

We fractionated each soil sample by material density to isolate three SOM pools: free POM (fPOM), occluded POM (oPOM), and MAOM as in Lang *et al*. (2023). Separating the POM into free and occluded pools allowed us to evaluate the impacts of root and leaf litter characteristics on the formation of large pieces of loosely integrated plant material in soil (fPOM) from those that have been contained within aggregates (oPOM). Briefly, soil samples were suspended in a solution of sodium polytungstate with a density of 1.7 g ml^−1^, centrifuged, and the free light particulate fraction was removed from the surface of the solution by aspiration. Next, occluded material was released from aggregates by shaking for 16 h at a speed of 200 oscillations per minute, and occluded POM was subsequently removed by aspiration. Organic matter remaining in the samples after isolating the particulate fractions was considered to be associated with the dense, or mineral soil fraction, and was classified as MAOM. All soil density fractions were thoroughly rinsed with nanopure water and oven dried before elemental analysis.

We determined the mass percentage of C and N as well as the δ^13^C and δ^15^N of all soil fractions, fine roots, and leaf litter (Supporting Information Table S1) using a Delta V Advantage mass spectrometer (Thermo‐Fisher Scientific, Waltham, MA, USA) coupled to an Elementar vario ISOTOPE Cube Elemental Analyzer (Elementar Americas Inc., Ronkonkoma, NY, USA). The resulting data were processed using Isodat 3.0 (Thermo‐Fisher Scientific). Analyses were conducted in the Stable Isotope Mass Spectrometry Laboratory at the Smithsonian Museum Conservation Institute in Suitland, MD, in 2021.

### Statistical analysis

First, we constructed simple linear models to test for relationships between above‐ and belowground biomass (i.e. stem basal area vs fine root mass) and litter input chemistry (litter C : N vs root C : N). We evaluated relationships between root and leaf litter C : N for each mixed‐species plot and at the individual species level. Plot‐level leaf and root C : N was determined by calculating a weighted average of the litter inputs using species‐specific tissue chemistry data (Table 1). We used basal area representation of each tree species to approximate the litter contribution of each species. Next, we investigated the potential for tree mycorrhizal association to act as an integrative trait explaining leaf and root litter chemistry and stem and root biomass, with the goal of using mycorrhizal type to predict tree species effects on POM‐C and MAOM‐C concentrations in soil and the contribution of trees to MAOM in our study plots. We constructed simple linear models to compare total stem basal area and fine root mass with the percentage of the basal area represented by AM‐associated tree species. We used linear mixed effects models with mycorrhizal type as a fixed effect and tree species as a random effect to evaluate the impact of mycorrhizal type on leaf litter C : N and fine root C : N. Linear mixed effects models were constructed using the lme4 package in R (Bates *et al*., 2015).

Next, we constructed a series of simple linear models to evaluate the effects of fine root tissue mass, stem basal area, root C : N, leaf C : N, and the percentage of AM‐associated tree basal area on the concentrations of soil C associated with each SOM fraction (fPOM, oPOM, and MAOM; mg C in each SOM fraction per gram of bulk soil) in the upper 5 cm of soil in each plot. Because our analysis was not focused on differences in SOM with depth, data from the 0 to 2 cm depth and from the 2 to 5 cm depth were combined to create a plot‐level value of SOM‐C concentration for all analyses. One plot with an exceptionally high MAOM‐C concentration (> 5 SD from mean value) was excluded from analyses of MAOM‐C concentration in soil but was included in subsequent isotope partitioning analyses because relative isotopic values of the sample were consistent with other plots.

Using a two‐pool isotope partitioning model, we determined the fraction of MAOM‐C derived from more recent plant inputs (i.e. the planted trees) and from the previous plant species at the site (corn; Fig. S1). Because the different photosynthetic pathways used by trees (C_3_) and corn (C_4_) result in distinct isotopic signatures of their residues, we used a two end‐member mixing model to determine the fraction of the MAOM‐C derived from the planted trees in each experimental plot, as below:

(Eqn 1) $$F\left(\text{MAOM}‐C_{\text{tree}}\right)=\left(\delta^{13}C_{\text{MAOM}}-n\right)/\left(\delta^{13}C_{\text{tree}}-n\right)$$

where δ^13^C_MAOM_ is the isotopic signature of the present‐day MAOM isolated in each plot, δ^13^C_tree_ is the δ^13^C of tree‐derived organic matter from each plot, and *n* represents the best available approximation of the preplanting MAOM δ^13^C. We conservatively estimated preplanting MAOM δ^13^C by selecting the least negative value in the current day samples (−14.876‰), which is consistent with the isotope ratio of corn‐planted soils (Keller *et al*., 2021). The δ^13^C_tree_ was calculated by taking the average δ^13^C of leaf and root litter from each plot. To investigate the individual roles of above‐ and belowground inputs on SOM pools, ideal data would allow us to partition root and leaf litter inputs with a dual‐isotope three‐source partitioning model (Phillips & Koch, 2002; Whitman & Lehmann, 2015). However, the δ^13^C and δ^15^N of the root and leaf tissues in this study were not distinct enough to allow us to conclusively separate the relative contributions of each tissue type to present‐day MAOM. Therefore, we used a two‐pool model to isolate corn‐derived MAOM‐C from tree‐derived MAOM‐C, and assumed an equal contribution of root and leaf litter to the tree‐derived MAOM when defining the tree‐derived C isotope ratio. By assuming an equal contribution of leaf and root tissue to the MAOM in our isotope partitioning model, we can use subsequent linear models to approximate the relative influence of each organic matter type on the formation of new MAOM‐C without introducing bias toward the importance of either above‐ or belowground inputs. To do so, we evaluated a series of simple linear models comparing the proportion of tree‐derived C in the MAOM fraction against plot‐level root C : N, leaf litter C : N, fine root mass, plot basal area, and the percentage of AM‐associated tree basal area. We then tested the impact of our assumption that root and leaf litter contributed equally to the isotope ratio of the tree‐derived MAOM by evaluating models with isotope ratios reflective of only root material and only leaf material; neither changed the significance of the relationships between tree‐derived MAOM content and the variables listed previously (Table S2). All analyses were conducted in R v.4.4.0 (R Core Team, 2018).

## Results

### Root and leaf litter properties

**Fig. 2:**
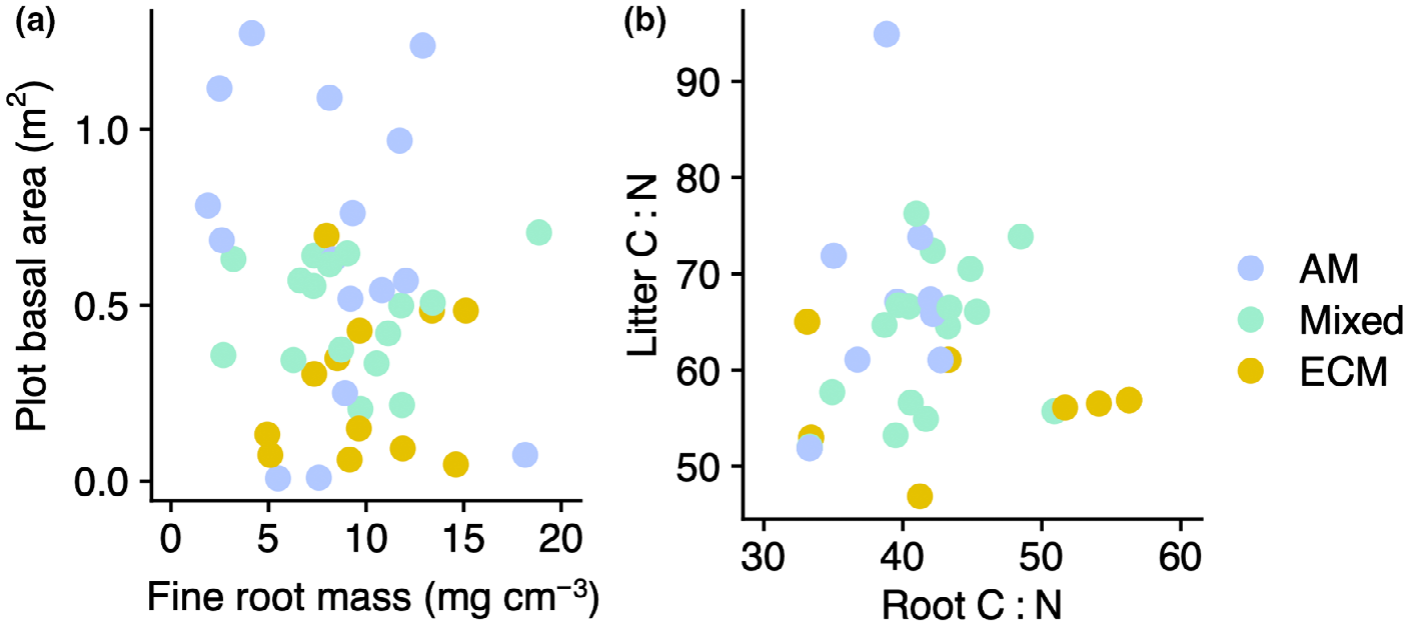
Relationships between above‐ and belowground tree biomass metrics and tissue chemistry. (a) Total plot basal area (m^2^) is not associated with fine root mass (mg cm^−3^) in each study plot. (b) Species‐weighted means of plot‐level leaf and root litter carbon to nitrogen ratios are not significantly related. Plot‐level mycorrhizal associations (AM, arbuscular mycorrhizal; ECM, ectomycorrhizal; Mixed, both arbuscular and ectomycorrhizal) were determined by basal area percentage, with AM and ECM plots having > 90% basal area from AM‐ and ECM‐associated trees, respectively. Plots dominated by AM tree species are shown in blue, mixed plots in green, and plots dominated by ECM trees in yellow.

Across the 43 study plots, the species‐weighted mean C : N of leaf litter ranged from 46.9 to 94.9 (mean: 63.1; SE: 1.5) and the species‐weighted mean C : N of fine roots ranged from 33.1 to 56.3 (mean: 41.4; SE: 1.0). The basal area of the trees in each plot was not associated with fine root mass in the upper 5 cm of soil (*F*_1,42_ = 1.1, *P* = 0.30; Fig. 2a). Furthermore, leaf C : N and root C : N were not correlated either at the community level in each plot (*F*_1,41_ = 0.08, *P* = 0.77; Fig. 2b) or within individual tree species (*F*_1,7_ = 0.07, *P* = 0.79). Plot mycorrhizal associations based on stem basal area ranged from 100% AM to 100% ECM‐associated tree species. Neither fine root C : N nor leaf litter C : N was related to the tree species' mycorrhizal association (*F* = 1.80, *P* = 0.45; *F* = 0.59, *P* = 0.190). Likewise, the percentage of AM‐associated tree basal area in each plot was not associated with fine root mass (*F*_1,42_ = 0.08, *P* = 0.77) but was positively associated with total stem basal area (*F*_1,42_ = 9.79, *P* = 0.003).

### POM‐C and MAOM‐C concentrations

**Fig. 3:**
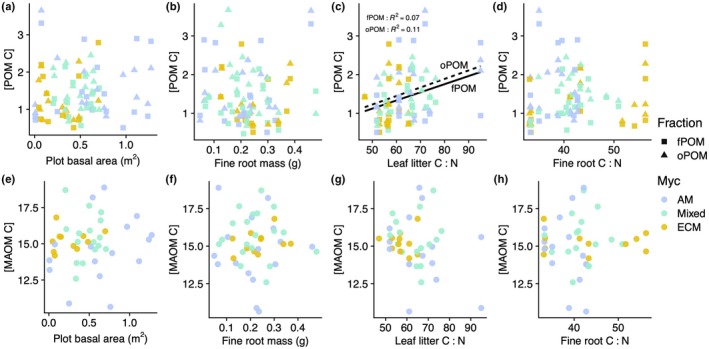
Soil organic matter (SOM) carbon concentrations with respect to tree biomass and tissue chemistry metrics. Concentrations of free particulate organic matter and occluded particulate organic matter C (panels a–d) and mineral‐associated organic C (MAOM, panels e–h; mg C g^−1^ soil) with respect to plot basal area (m^2^), fine root mass (g), and plot‐level averages of leaf litter and fine root carbon : nitrogen ratio (C : N). Each SOM fraction was isolated from a soil sample taken from the top 5 cm of soil at the center of each experimental plot. Leaf and root litter C : N was determined as the average of the C : N of litter from species present in each plot, weighted by species basal area. Linear models are shown only for plots with significant relationships among variables. Plots dominated by arbuscular mycorrhizal tree species are shown in blue, mixed plots in green, and plots dominated by ectomycorrhizal trees in yellow.

About 84.1% of the total SOM‐C was contained in the MAOM fraction, 7.3% in the fPOM fraction, and 8.6% in the oPOM fraction. The concentrations of SOM‐C associated with each density fraction were not related to either plot basal area or fine root litter mass (Fig. 3a,b,e,f; Table S3). The concentrations of fPOM‐C and oPOM‐C (mg SOM‐C g^−1^ soil) were positively associated with plot‐level leaf litter C : N (Fig. 3c; fPOM: *F*_1,41_ = 3.93, *P* = 0.054; oPOM: *F*_1,41_ = 6.35, *P* = 0.016), but not associated with root C : N (Fig. 3d). MAOM‐C concentration was not impacted by either leaf or root litter chemistry (Fig. 3g,h). Two plots with monocultures of *Liquidambar styraciflua* had leaf litter C : N ratios that were 50% higher than the mean litter C : N across all plots (Table 1), and the effect of leaf litter C : N on fPOM‐C and oPOM‐C concentrations in soil was slightly weaker with these plots excluded from the analysis (Table S4). The basal area percentage of AM‐associated trees was unrelated to concentrations of C in any SOM fraction (Fig. 3a,e; Table S3).

### Contribution of tree‐derived C to MAOM

**Fig. 4:**
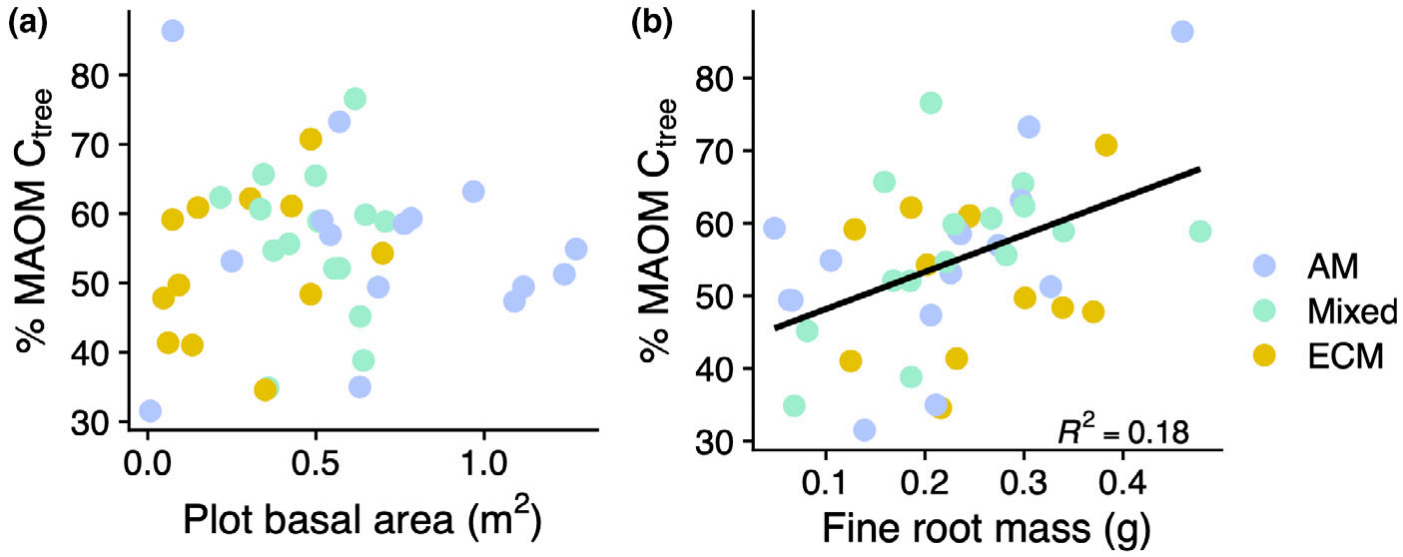
Factors associated with the percentage of the mineral‐associated organic matter carbon (MAOM‐C) derived from tree tissue. (a) Tree‐derived MAOM‐C is not related to plot‐level basal area, but is positively related to the fine root mass in the upper 5 cm of soil (b; *F*_1,40_ = 10.1, *P* = 0.003; *n* = 42 plots). Each point represents one plot (*n* = 42). Plots dominated by arbuscular mycorrhizal tree species are shown in blue, mixed plots in green, and plots dominated by ectomycorrhizal trees in yellow.

Between 32% and 86% of the C in the MAOM fraction had an isotope signature indicating it was derived from the planted trees rather than corn. The fraction of tree‐derived MAOM‐C was not affected by the biomass of trees in each plot as estimated with stem basal area, nor with the basal area percentage of AM‐associated tree species (Table S5), but was higher in plots with greater fine root mass in the upper 5 cm of soil (*F*_1,40_ = 10.1, *P* = 0.003; Fig. 4). Neither root nor leaf litter C : N was associated with the fraction of tree‐derived MAOM‐C (Table S5).

## Discussion

We expected tree biomass would be positively associated with the concentrations of C in all SOM fractions due to higher rates of plant tissue production and litter inputs to soil. Instead, we found no effect of either plot basal area or fine root mass on total SOM‐C concentrations (Fig. 3). This pattern may be a result of two competing effects of tree productivity: the production of litter inputs to soil and the increasing activity of fine roots. Root exudates are known to act as priming agents for SOM decomposition (Kuzyakov *et al*., 2000; Jackson *et al*., 2019; Lang *et al*., 2020) and thus may offset litter‐derived C additions by promoting the decomposition of existing SOM (Lajtha *et al*., 2018; Li *et al*., 2019; Dijkstra *et al*., 2021; Huang *et al*., 2021; Pierson *et al*., 2021). Therefore, when rapid plant growth leads to high nutrient demand, the activity of fine roots may increase in concert with plant litter production and rhizodeposition, effectively resulting in no net change to the concentrations of SOM‐C with increasing plant productivity. This root priming effect on SOM‐C may be diminished over time, as forests mature and individual tree growth rates slow, eventually leading to accumulation of C in POM and MAOM (i.e. on the order of decades (Cunningham *et al*., 2015)).

We also expected organic matter chemistry would influence the formation of POM and MAOM, but that root inputs would be more closely associated with MAOM‐C and leaf inputs with POM‐C. In partial support of this hypothesis, we found that leaf litter chemistry, but not root chemistry, was associated with the production of both free and occluded POM (Fig. 3). In agreement with results of other studies (Cyle *et al*., 2016; Almeida *et al*., 2021), low‐quality leaf litter (i.e. high C : N) tended to favor the accumulation of C in the POM fraction. Leaf litter C : N, however, did not impact the concentration of MAOM‐C in the soil, suggesting that leaf inputs do not constitute a major component of MAOM in this ecosystem. Likewise, given that neither fine root biomass nor C : N was associated with POM‐C concentrations, we suggest that roots are not primary contributors to POM‐C in the BiodiversiTREE plots. The importance of leaf, but not root litter, as a driver of SOM‐C has been noted in other temperate forests (Bowden *et al*., 2014; Li *et al*., 2019; Cao *et al*., 2020) and may be related to stand age. The BiodiversiTREE plots were 7 yr at the time of soil sampling for this study, and therefore likely to exhibit tree effects on SOM that differ from those described in mature forests. Smaller trees tend to allocate a relatively larger fraction of their total biomass to leaf tissue and a smaller fraction to roots, compared with larger trees (Poorter *et al*., 2012; Smith‐Martin *et al*., 2020), which may result in a greater proportion of leaf litter vs root litter inputs to soil in young forests. Additionally, low root mortality in young stands may limit rates of root litter production and contributions to POM‐C (Yuan & Chen, 2012). Lastly, given that we found no relationships between tree mycorrhizal association and leaf litter C : N or fine root C : N, it is perhaps unsurprising that plot mycorrhizal type was unrelated to the concentrations of both POM‐C and MAOM‐C (Fig. 3).

The patterns we found in the fraction of tree‐derived MAOM‐C provide further evidence that root inputs are the primary constituents of recently formed MAOM. With increasing fine root mass, more of the MAOM‐C had an isotope signature indicating it was derived from trees. Importantly, this increase in the MAOM‐C_tree_ fraction was associated only with root mass, but not aboveground plot productivity (Fig. 4). Together, these results indicate that it is the root biomass in particular, and not general metrics of tree productivity, that may be used to predict rates of MAOM formation. Positive effects of fine roots on stable SOM formation have also been demonstrated under experimental conditions in mature temperate forests (Crow *et al*., 2009b; Bowden *et al*., 2024), indicating that this effect may be consistent across forests of different age classes. Furthermore, because root C : N was not an important determinant of the fraction of MAOM‐C_tree_, these patterns add to the evidence suggesting that the activity of live roots (i.e. exudation of low‐molecular‐weight organic compounds, rhizodeposits and production of root‐associated fungal hyphae), rather than root tissue turnover, is a primary driver of MAOM formation (Sokol *et al*., 2019; Villarino *et al*., 2021; Fahey *et al*., 2023). However, coupled with no increase in the total concentration of MAOM‐C along a gradient of root biomass, our work shows that live root inputs may not necessarily lead to net soil C accumulation in mineral‐associated forms. These patterns add further support for the idea that priming by live roots offsets root‐derived MAOM formation, resulting in a relatively stable MAOM pool size with a high C turnover rate.

Despite reports that AM‐associated tree roots decay faster (See *et al*., 2019; Beidler *et al*., 2023) and promote more MAOM‐C formation than do ECM roots (Craig *et al*., 2018; Cotrufo *et al*., 2019; Keller *et al*., 2021), we did not find that plot mycorrhizal type impacted the root contribution to MAOM‐C_tree_. This may be the result of several factors. First, the root exudation rate of the particular tree species planted in our study plots may not have been systematically different with respect to mycorrhizal type, a pattern also noted elsewhere (Liese *et al*., 2018; Chari *et al*., 2024; Oh *et al*., 2024). Our study included only a small number of species within each mycorrhizal group, and the traits of these species may not represent the general trends within these functional groups. Therefore, we cannot confidently attribute patterns in our results to differences in mycorrhizal type, but rather acknowledge the likely large influence of individual species traits in the formation of new MAOM (Angst *et al*., 2019). Plots with relatively more AM‐associated trees, however, did tend to have greater overall basal area, likely due to the high growth rates of the particular AM species included in the BiodiversiTREE study. Furthermore, the trees in the study area may not be established enough to show differences in MAOM formation with mycorrhizal type; given reports of mycorrhizal effects from older common gardens and synthesis studies (Peng *et al*., 2020; Rożek *et al*., 2023), it is possible that over time, distinctions between root contributions to MAOM in AM‐dominated plots and ECM‐dominated plots will emerge at the Smithsonian Environmental Research Center BiodiversiTREE site.

While our results suggest that the activity of fine roots is associated with MAOM‐C formation, our ability to definitively rule out the potential contributions of leaf litter chemistry and biomass, and root litter chemistry, was limited by our study design. First, we used the chemistry of fine roots isolated from our soil samples to represent the composition of fine root litter, although the samples contained both live and dead roots. Roots that were living at the time of sampling likely had a lower C : N, which could have reduced the variability in root litter chemistry across the study plots and masked potential patterns in root litter contributions to MAOM‐C. Furthermore, our metrics of leaf litter chemistry were based on aggregated litter samples, which may not reflect plot‐to‐plot variation in litter chemistry that can arise due to environmental and competitive conditions. Aboveground biomass, estimated with total tree basal area, is likewise not a direct measure of leaf litter input, and species‐specific differences in litter production vs stem diameter are likely. All together, these proxy measures of leaf and root litter characteristics may not have allowed us to detect the effects of these organic matter inputs on MAOM‐C formation, and we suggest that continued work with more targeted sampling of these SOM variables would clarify their contribution to stable soil C. Additionally, effects of soil fauna were not assessed in this study, although it is known that earthworms are present at the SERC site (Szlávecz & Csuzdi, 2007; Crow *et al*., 2009a). Earthworms can influence both POM and MAOM pools through bioturbation, displacing surface litter, consuming roots, and depositing mucilage on soil minerals (Angst *et al*., 2022; Zhao *et al*., 2025). We assumed an equal influence of earthworms, and soil fauna more generally, across our study plots, but this was not confirmed with sampling and could have led to bias in our SOM‐C data. Lastly, minerals in surface soils can become saturated with organic matter, which, if it occurred in our site, would limit our ability to detect ongoing changes associated with new organic matter inputs. Given that MAOM is increasingly recognized as an actively cycling, though relatively stable pool of soil C, the possibility of mineral saturation is further complicated by uncertainty about the turnover of various forms of MAOM‐C (Jilling *et al*., 2025).

### Conclusion

Overall, our results support the hypothesis that MAOM formation is impacted by root inputs more directly than by leaf inputs. Conversely, patterns in particulate C concentration indicate that leaf litter chemistry, but not root litter chemistry, influences POM‐C pools. These findings reinforce the existing theory that live roots are the primary contributors to MAOM but that their effects on net C sequestration in soil may be limited due to the priming effect of root activity. Furthermore, the lack of expected differences in SOM fractions with tree mycorrhizal type may indicate that mycorrhizal effects are not apparent in young (< 10 yr old) stands or with the particular species studied here. Our work highlights the distinct controls on POM and MAOM pools and underscores the importance of studying these fractions individually to best represent soil C dynamics in forest ecosystems.

## Competing interests

None declared.

## Author contributions

JDP and RPP designed the experiment and provided the initial motivation for this work; CF, JP and RAK conducted fieldwork and sampling; CF and JDP provided ancillary data; AL, JP and RAK conducted the laboratory analyses; AL conducted statistical analyses and wrote the manuscript. All authors contributed substantially to revising the manuscript.

## Disclaimer

The New Phytologist Foundation remains neutral with regard to jurisdictional claims in maps and in any institutional affiliations.

## Supporting information


**Fig. S1** Carbon isotope ratios (δ^13^C) of each plant tissue sample and soil organic matter fraction.
**Table S1** δ^13^C of all soil fractions, fine roots, and leaf litter from the experimental plots and tree species used in this study.
**Table S2** Hypothetical assessment of the relationships between fine root mass and the proportion of tree‐derived MAOM C, with isotope ratios adjusted to account for possible bias in the assumption of equal leaf and root litter contribution.
**Table S3** Results of linear models for the effects of fine root mass, plot basal area, fine root C : N, and leaf litter C : N on the concentrations of C in each SOM fraction per gram of bulk soil (mg SOM g^−1^ soil).
**Table S4** Results of linear models for the effect of leaf litter C : N on the concentrations of C in each SOM fraction per gram of bulk soil (mg SOM g^−1^ soil) with *Liquidambar styraciflua* monocultures (*n* = 2 plots) excluded from the analysis due to high litter C : N.
**Table S5** Results of linear models for the effects of fine root mass, plot basal area, fine root C : N, and leaf litter C : N on the proportion of tree‐derived C in the MAOM fraction.
Please note: Wiley is not responsible for the content or functionality of any Supporting Information supplied by the authors. Any queries (other than missing material) should be directed to the *New Phytologist* Central Office.

## Data Availability

Data and code associated with this work are available through Figshare from the Smithsonian Institution, doi: 10.25573/serc.27015511.
