# Supplementary material for: Leaf litter and fine roots have distinct effects on particulate and mineral‐associated soil organic matter in a tree common garden
Source: New Phytol. 2026 Jan 5;249(5):2263–72. doi: 10.1111/nph.70854 (PMC12873499; doi:10.1111/nph.70854)

New Phytologist Supporting Information

Article title: **Leaf litter and fine roots have distinct effects on particulate and mineral-associated soil organic matter in a tree common garden**

Authors: Ashley Lang, Rachel A. King, Jamie Pullen, Catherine Fahey, John D. Parker, Richard P. Phillips

Article acceptance date: 24 November 2025

Table S1: $\delta^{13}C$ of all soil fractions, fine roots, and leaf litter from the experimental plots and tree species used in this study. Fine root samples were manually isolated from the soil samples prior to density fractionation. Freshly fallen leaf litter samples were collected from four plots containing monocultures of each tree species, with the exception of *Cornus florida*, which was collected from one plot. Leaf litter $\delta^{13}C$ represents the mean of all species $\delta^{13}C$ weighted by basal area representation in the plot. Root litter $\delta^{13}C$ quantified using the fine roots collected at the center of each plot. fPOM, oPOM, and MAOM $\delta^{13}C$ are means of each sampling depth (0-2, 2-5 cm). NR indicates natural regeneration (unplanted plots) which did not have foliage or root samples collected. NA indicates no data available. fPOM= free particulate organic matter, oPOM= occluded particulate organic matter, and MAOM= mineral-associated organic matter. CACA= *Carpinus caroliniana* Walt., COFL= *Cornus florida* L., FAGR= *Fagus grandifolia* Ehrh. Little, LIST= *Liquidambar stryaciflua* L., LITU= *Liriodendron tulipifera* L., PLOC= *Platanus occidentalis* L., QUAL= *Quercus alba* L., QUPA= *Quercus pagoda* Raf., and QURU= *Quercus rubra* L.

Plot	Diversity	Species	Leaf litter $\delta^{13}C$	Root litter $\delta^{13}C$	fPOM $\delta^{13}C$	oPOM $\delta^{13}C$	MAOM $\delta^{13}C$
1	1	PLOC	-28.52	-28.43	-28.31	-27.56	-21.6
3	1	QUPA	-30.86	-28.98	-29.19	-28.45	-23.04
4	4		-29.27	-28.49	-27.95	-26.68	-19.78
5	12		-29.71	-28.61	-25.94	-27.98	-22.32
10	12		-30.45	-28.91	-26.81	-28.98	-24.57
12	1	COFL	-29.59	-26.28	-25.08	-23.9	-18.99
13	12		-29.27	-28.63	-26.5	-28.27	-22.89
14	1	FAGR	-30.87	-29.28	-29.29	-27.3	-21.16
16	1	CACA	-31.4	-25.95	-26.07	-24.73	-20.54

17	12		-30.59	-29.01	-28.89	-26.2	-20.08
19	1	QUAL	-29.51	-25.85	-27.06	-26.34	-21
21	12		-29.83	-28.96	-26.86	-26.03	-20.52
22	4		-30.7	-28.81	-28.5	-27.24	-21.6
23	1	QUAL	-29.51	-25.85	-26.37	-26.97	-22.45
24	NR		NA	NA	-29.52	-28.4	-23.7
25	1	QURU	-30.06	-28.13	-25.45	-27.29	-23.71
26	12		-27.74	-26.76	-27.61	-27.68	-21.33
27	4		-29.5	-28.85	-27.08	-26.41	-21.65
28	1	LITU	-29.69	-29.13	-29.03	-27.71	-22.33
30	4		-30.86	-28.98	-25.71	-26.21	-20.08
31	1	LIST	-30.51	-28.6	-29.22	-28.05	-22.93
32	1	NYSY	NA	NA	-28.06	-27.05	-22.54
33	12		-29.34	-28.64	-29.14	-27.83	-23.19
36	12		-29.83	-28.78	-29.3	-28.16	-22.9
38	12		-29.33	-28.52	-28.57	-25.78	-14.88
40	1	COFL	-29.59	-26.28	-29.48	-29.68	-26.15
41	4		-28.52	-28.43	-27.99	-27.76	-24.84
42	12		-29.86	-28.81	-28.79	-29.25	-25.94
43	1	LIST	-30.51	-28.6	-28.46	-28.26	-22.68
44	1	QURU	-30.06	-28.13	-28.61	-27.65	-23.56
45	4		-29.69	-29.13	-28.4	-28.52	-24.06
47	4		-28.89	-28.46	-28.83	-28.67	-23.06
48	12		-28.8	-28.45	-27.88	-26.7	-21.67
49	1	FAGR	-30.87	-29.28	-25.72	-26.94	-22.43
51	NR		NA	NA	-25.85	-29.13	-23.79
52	4		-30.7	-28.8	-28.5	-27.32	-22.07

53	12		-29.36	-28.74	-28.55	-27.48	-23.23
54	4		-29.74	-28.98	-29.14	-27.55	-22.8
55	12		-29.41	-28.5	-27.7	-27.73	-23.66
60	12		-29.73	-28.78	-27.95	-27.9	-23.6
62	1	PLOC	-28.52	-28.43	-27.72	-27.63	-22.84
63	1	QUPA	-30.86	-28.98	-28.83	-27.72	-25.52
64	4		-30.78	-28.89	-27.36	-27.22	-23.99
71	NR		NA	NA	-26.92	-28.71	-24.19
72	12		-29.69	-28.7	-29.1	-28.96	-24.28
73	1	LITU	-29.69	-29.13	-27.12	-27.96	-23.45
74	4		-30	-29.08	-27.25	-28.02	-23.66

Table S2: Hypothetical assessment of the relationships between fine root mass and the proportion of tree-derived mineral-associated organic matter carbon (MAOM-C), with isotope ratios adjusted to account for possible bias in the assumption of equal leaf and root litter contribution. Column 1 indicates the model results if all tree-derived organic matter was originally root material, while column 2 indicates model results if all tree-derived organic matter was originally leaf material. These results do not vary substantially from the results of the model using the mean value of the leaf and root litter isotope ratios. AM=arbuscular mycorrhizal.

Parameter	F(MAOM C_{tree}): all root material		F(MAOM C_{tree}): all leaf material	
Fine root mass	$F_{1,40}$= 10.02	p=0.003	$F_{1,40}$= 10.17	p=0.003
Plot basal area	$F_{1,40}$= 0.02	p=0.90	$F_{1,40}$= 0.03	p=0.87
Fine root C:N	$F_{1,40}$= 0.50	p=0.49	$F_{1,40}$= 0.42	p=0.52
Leaf litter C:N	$F_{1,40}$= 0.05	p=0.83	$F_{1,40}$= 0.04	p=0.84
% AM basal area	$F_{1,40}$= 1.47	p=0.23	$F_{1,40}$= 1.35	p=0.25

Table S3: Results of linear models for the effects of fine root mass, plot basal area, fine root C:N, and leaf litter C:N on the concentrations of C in each SOM fraction per gram of bulk soil (mg SOM g soil). Each SOM fraction was isolated from a soil sample taken from the top 5 cm of soil at the center of each experimental plot. Leaf and root litter C:N was determined as the average of the C:N of litter from species present in each plot, weighted by species representation. fPOM= free particulate organic matter, oPOM= occluded particulate organic matter, and MAOM= mineral-associated organic matter.

**Parameter**	**[fPOM C]**		**[oPOM C]**		**[MAOM C]**	
Fine root mass	$F_{1,45}$= 0.001	p=0.97	$F_{1,45}$= 0.0005	p=0.98	$F_{1,44}$ = 0.007	p=0.93
Plot basal area	$F_{1,42}$= 1.06	p=0.31	$F_{1,42}$= 0.001	p=0.97	$F_{1,41}$= 0.53	p=0.47
Fine root C:N	$F_{1,41}$= 0.59	p=0.44	$F_{1,41}$= 0.01	p=0.94	$F_{1,40}$= .001	p=0.97
Leaf litter C:N	$F_{1,41}$= 3.93	p=0.054	$F_{1,41}$= 6.35	p=0.016	$F_{1,40}$ = 1.78	p=0.19
% AM Basal area	$F_{1,42}$= 0.73	p=0.40	$F_{1,42}$= 2.10	p=0.16	$F_{1,421}$= 0.59	p=0.44

Table S4: Results of linear models for the effect of leaf litter C:N on the concentrations of C in each soil organic matter (SOM) fraction per gram of bulk soil (mg SOM g soil) with *Liquidambar styraciflua* monocultures (n=2 plots) excluded from the analysis due to high litter C:N. Each SOM fraction was isolated from a soil sample taken from the top 5 cm of soil at the center of each experimental plot. Leaf and root litter C:N was determined as the average of the C:N of litter from species present in each plot, weighted by species representation. fPOM= free particulate organic matter, oPOM= occluded particulate organic matter, and MAOM= mineral-associated organic matter.

Parameter	[fPOM C]	[oPOM C]	[MAOM C]
Leaf litter C:N	$F_{1,39}$= 3.37 *p*= 0.07	$F_{1,39}$= 2.91 *p*= 0.09	$F_{1,38}$= 0.09 *p*= 0.77

Table S5: Results of linear models for the effects of fine root mass, plot basal area, fine root C:N, and leaf litter C:N on the proportion of tree-derived carbon (C) in the mineral-associated organic matter (MAOM) fraction. MAOM was isolated from a soil sample taken from the top 5 cm of soil at the center of each experimental plot. Leaf and root litter C:N was determined as the average of the C:N of litter from species present in each plot, weighted by species representation. Proportion MAOM from tree-derived C was determined with a two-end member isotope mixing model. MAOM C from trees was distinguished from MAOM C derived from corn, the former dominant vegetation in the study site.

**Parameter**	**F(MAOM C_{tree})**	
Fine root mass	$F_{1,40}$ = 10.1	p=0.003
Plot basal area	$F_{1,40}$ = 0.02	p=0.88
Fine root C:N	$F_{1,40}$ = 0.45	p=0.50
Leaf litter C:N	$F_{1,40}$ = 0.04	p=0.83
% AM basal area	$F_{1,40}$ = 1.40	p=0.24

Figure S1: Carbon isotope ratios ($\delta^{13}C$) of each plant tissue sample and soil organic matter fraction. Central bars indicate mean values and error bars represent standard error of the mean.

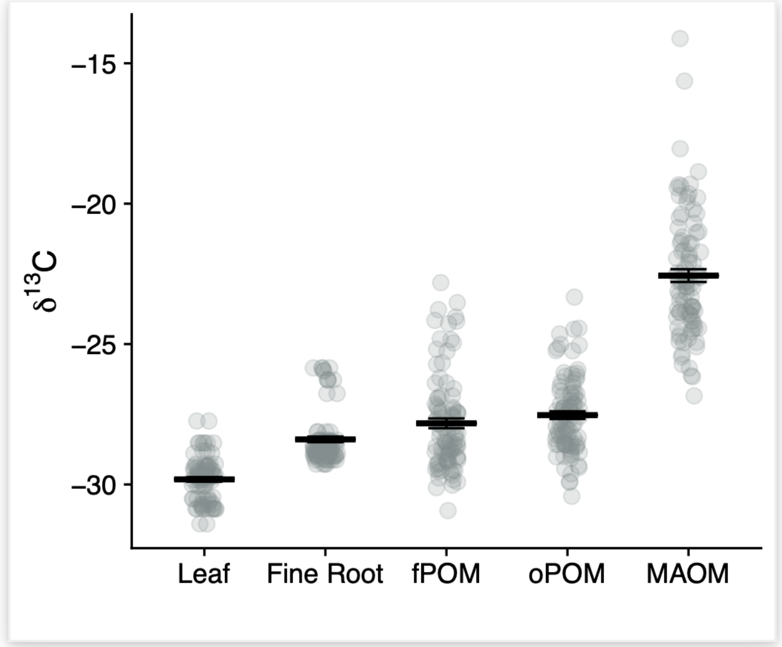

Supplement: Supplementary file 1 — Fig. S1 Carbon isotope ratios (δ13C) of each plant tissue sample and soil organic matter fraction. Table S1 δ13C of all soil fractions, fine roots, and leaf litter from the experimental plots and tree species used in this study. Table S2 Hypothetical assessment of the relationships between fine root mass and the proportion of tree‐derived MAOM C, with isotope ratios adjusted to account for possible bias in the assumption of equal leaf and root litter contribution. Table S3 Results of linear models for the effects of fine root mass, plot basal area, fine root C : N, and leaf litter C : N on the concentrations of C in each SOM fraction per gram of bulk soil (mg SOM g−1 soil). Table S4 Results of linear models for the effect of leaf litter C : N on the concentrations of C in each SOM fraction per gram of bulk soil (mg SOM g−1 soil) with Liquidambar styraciflua monocultures (n = 2 plots) excluded from the analysis due to high litter C : N. Table S5 Results of linear models for the effects of fine root mass, plot basal area, fine root C : N, and leaf litter C : N on the proportion of tree‐derived C in the MAOM fraction. Please note: Wiley is not responsible for the content or functionality of any Supporting Information supplied by the authors. Any queries (other than missing material) should be directed to the New Phytologist Central Office. [file NPH-249-2263-s001.pdf]
